# Spatiotemporal trends and ecological determinants in maternal mortality ratios in 2,205 Chinese counties, 2010–2013: A Bayesian modelling analysis

**DOI:** 10.1371/journal.pmed.1003114

**Published:** 2020-05-15

**Authors:** Junming Li, Juan Liang, Jinfeng Wang, Zhoupeng Ren, Dian Yang, Yanping Wang, Yi Mu, Xiaohong Li, Mingrong Li, Yuming Guo, Jun Zhu

**Affiliations:** 1 School of Statistics, Shanxi University of Finance and Economics, Taiyuan, Shanxi, China; 2 National Office for Maternal and Child Health Surveillance of China, Department of Obstetrics, West China Second University Hospital, Sichuan University, Chengdu, Sichuan, China; 3 State Key Laboratory of Resources and Environmental Information System (LREIS), Institute of Geographic Sciences and Natural Resources Research, Chinese Academy of Sciences, Beijing, China; 4 University of Chinese Academy of Sciences, Beijing, China; 5 School of Public Health and Preventive Medicine, Monash University, Melbourne, Australia; International Rice Research Institute, PHILIPPINES

## Abstract

**Background:**

As one of its Millennium Development Goals (MDGs), China has achieved a dramatic reduction in the maternal mortality ratio (MMR), although a distinct spatial heterogeneity still persists. Evidence of the quantitative effects of determinants on MMR in China is limited. A better understanding of the spatiotemporal heterogeneity and quantifying determinants of the MMR would support evidence-based policymaking to sustainably reduce the MMR in China and other developing areas worldwide.

**Methods and findings:**

We used data on MMR collected by the National Maternal and Child Health Surveillance System (NMCHSS) at the county level in China from 2010 to 2013. We employed a Bayesian space–time model to investigate the spatiotemporal trends in the MMR from 2010 to 2013. We used Bayesian multivariable regression and GeoDetector models to address 3 main ecological determinants of the MMR, including per capita income (PCI), the proportion of pregnant women who delivered in hospitals (PPWDH), and the proportion of pregnant women who had at least 5 check-ups (PPWFC). Among the 2,205 counties, there were 925 (42.0%) hotspot counties, located mostly in China’s western and southwestern regions, with a higher MMR, and 764 (34.6%) coldspot counties with a lower MMR than the national level. China’s westernmost regions, including Tibet and western Xinjiang, experienced a weak downward trend over the study period. Nationwide, medical intervention was the major determinant of the change in MMR. The MMR decreased by 1.787 (95% confidence interval [CI]: 1.424–2.142, *p* < 0.001) per 100,000 live births when PPWDH increased by 1% and decreased by 0.623 (95% CI 0.436–0.798, *p* < 0.001) per 100,000 live births when PPWFC increased by 1%. The major determinants for the MMR in China’s western and southwestern regions were PCI and PPWFC, while that in China’s eastern and southern coastlands was PCI. The MMR in western and southwestern regions decreased nonsignificantly by 1.111 (95% CI −1.485–3.655, *p* = 0.20) per 100,000 live births when PCI in these regions increased by 1,000 Chinese Yuan and decreased by 1.686 (95% CI 1.275–2.090, *p* < 0.001) when PPWFC increased by 1%. Additionally, the western and southwestern regions showed the strongest interactive effects between different factors, in which the corresponding explanatory power of any 2 interacting factors reached up to greater than 80.0% (*p* < 0.001) for the MMR. Limitations of this study include a relatively short study period and lack of full coverage of eastern coastlands with especially low MMR.

**Conclusions:**

Although China has accomplished a 75% reduction in the MMR, spatial heterogeneity still exists. In this study, we have identified 925 (hotspot) high-risk counties, mostly located in western and southwestern regions, and among which 332 counties are experiencing a slower pace of decrease than the national downward trend. Nationally, medical intervention is the major determinant. The major determinants for the MMR in western and southwestern regions, which are developing areas, are PCI and PPWFC, while that in China’s developed areas is PCI. The interactive influence of any two of the three factors, PCI, PPWDH, and PPWFC, in western and southwestern regions was up to and in excess of 80% (*p* < 0.001).

## Abstract 2

Junming Li and co-workers report on trends in maternal mortality at county level across China.

## Author summary

**Why was this study done?:**

- Information about the spatiotemporal trends of the maternal mortality ratio is helpful in the policymaking response to reducing the maternal mortality ratio (MMR) in developing areas.
- The study can help the government to preassess the effects of policy if the corresponding magnitudes of influence of the underlying determinants can be quantified.
- The quantitative statistical results of national and subnational influencing effects and patterns can help the government to create policies with precision.

**What did the researchers do and find?:**

- We employed a Bayesian space–time model to explore the spatiotemporal trends of the MMR in 2,205 Chinese counties from 2010 to 2013 and used Bayesian multivariable regression and GeoDetector models to address 3 main ecological determinants of MMR.
- The major determinants of the MMR in China are medical intervention factors. The MMR will decrease by 1.787 (95% CI 1.424–2.142, *p* < 0.001) and 0.623 (95% CI 0.436–0.798, *p* < 0.001) per 100,000 live births when the proportion of hospital births and the proportion of 5 or more antenatal care visits increase by 1.0%.
- The major determinants for the MMR in the western and southwestern regions of China are per capita income and antenatal care, while in the eastern and southern coastal regions, it is per capita income.

**What do these results mean?:**

- Many countries, and particularly developing countries, may learn from China’s dramatic improvement in maternal survival rates.
- This progress has profited from long-term strategies to enhance delivery care in healthcare facilities and the provision of professional maternity care in large hospitals. There are, however, a variety of policy effects that have occurred in different areas due to regional heterogeneity.
- We have revealed the dominant factors and their corresponding influencing magnitudes at the national and subnational level, and this evidence may help China or other developing countries to preassess policy effects.

## Introduction

Although some countries have achieved a decrease in the maternal mortality ratio (MMR), maternal mortality remains a global public health issue [1]. It is estimated that roughly 303,000 maternal deaths occurred worldwide in 2015, mostly in low-income and middle-income countries, and most of which could have been prevented [2]. As the most populous developing country in the world, the maternal mortality trend in China has a significant impact on that of the world. According to a report [3], nearly 86% of maternal deaths in China may be avoidable. Reducing maternal mortality is an aspiration of the international community, and the issue has been widely studied [1,4–6].

China has more than 17 million live births each year and in 2014 reached the target of the fifth of its Millennium Development Goals (MDG 5) by achieving a 75% reduction in the MMR (the number of maternal deaths per 100,000 live births) from 1990 to 2015 [1]. Some regions, however, especially those in the northwest, still have far to go in reaching this target [1,7]. China has also achieved the United Nations Sustainable Development Goals (SDGs) target of reducing the MMR to less than 70.0 per 100,000 live births [1]. Although China’s maternal mortality declined from 111.0 per 100,000 live births in 1990 to 21.8 per 100,000 live births in 2015 [1], some counties of western China have not achieved this level [8], and the absolute number of maternal deaths in China still has a significant impact on the global MMR. Avoidable deaths account for 60%–70% of maternal mortality, which means that there is still room for reducing the figure in China.

China has a spatially stratified heterogeneity in medical technology level, economy, culture, and geographic environment. These differences affect the occurrence of and deaths from childbirth [9,10]; sometimes, the influence of these factors far exceeds the treatment itself, but their roles are often overlooked. Effective intervention in maternal mortality requires a better understanding of the exact geographical distribution and its impact on MMR. We explored the spatiotemporal trends of China’s MMR over the period 2010–2013 and estimated the relative risks in different 2,205 counties. We also measured the relationship between the MMR and its potential determinants or their proxies, including per capita income (PCI), antenatal care (using the proportion of antenatal care at the county level), and hospital delivery (using the proportion of pregnant women who delivered their babies in hospitals).

## Methods

### Study design

The target of this paper was to investigate the spatiotemporal trends in the MMR at the local (county) level in China from 2010 to 2013 and to address, nationally as well as subnationally, the main nonmedical impact factors on the MMR. Based on the maternal mortality data covering 1,832 counties across 23 provincial regions, excluding the 11 eastern coastal provincial regions, the spatiotemporal trends in the MMR for 2,205 Chinese counties were explored through a Bayesian space–time model. Among the 2,205 counties, the data surveillance occurred in 1,832 counties, while there were no survey data for the other 373 counties. Because of a full lack of sampling, the counties located in the 11 eastern coastal provincial regions of China (blank areas in S1 Fig) were not included in this study. The magnitude and patterns of influence of the main determinant factors were estimated using the Bayesian multivariable regression model and GeoDetector models.

**Fig 1:**
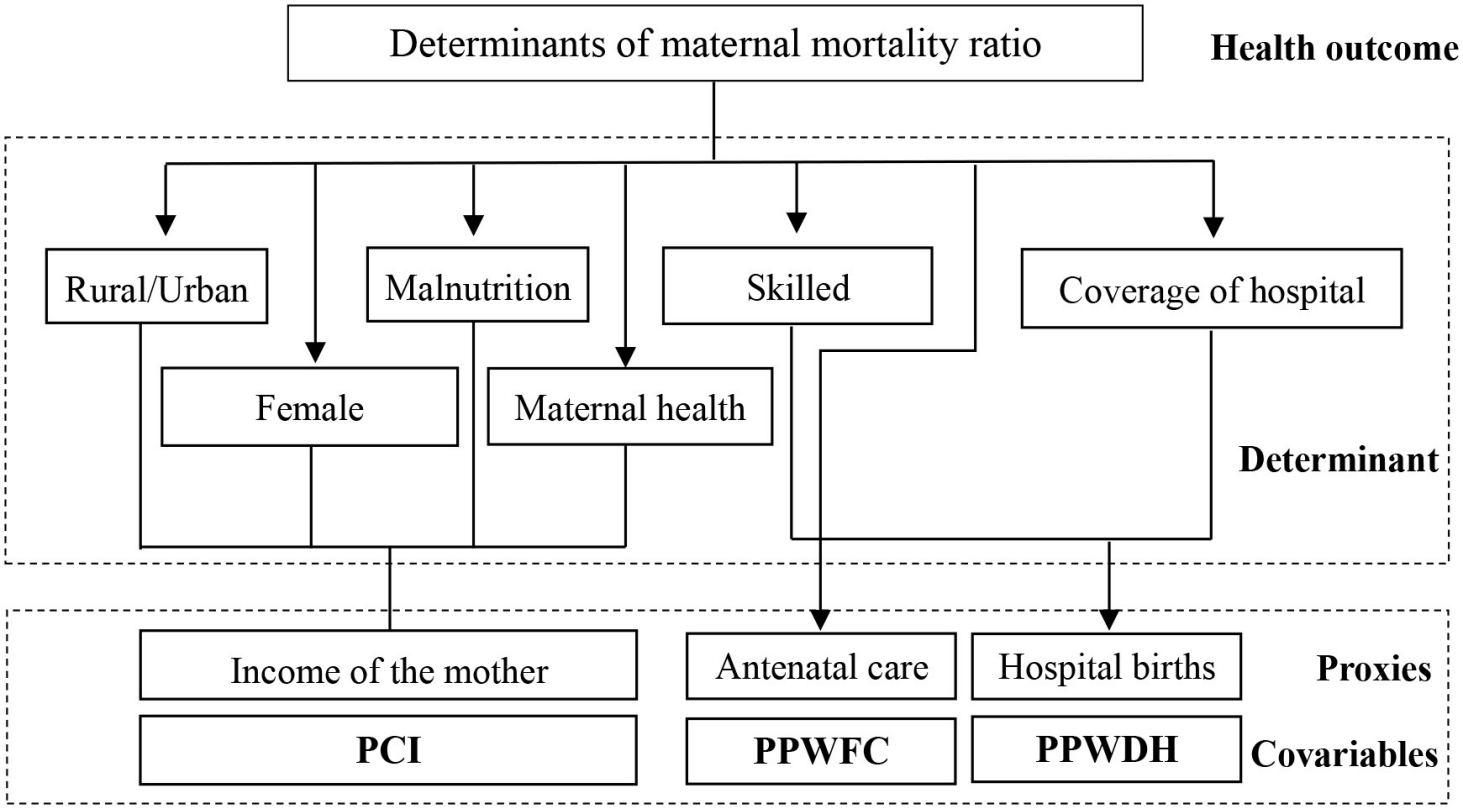
Nonmedical determinants (and their proxies) of the MMR. MMR, maternal mortality ratio; PCI, per capita income; PPWDH, proportion of pregnant women who delivered in hospitals; PPWFC, proportion of pregnant women who received 5 or more maternal check-ups.

The determinants of the MMR mainly include direct obstetric causes and indirect causes [11–13]. In this research, we do not analyse the medical determinants of the MMR, but rather focus on nonmedical determinants. One of the most important indirect factors is the mother’s income, which is a proxy of maternal health, education, malnutrition, and place of residence (urban or rural) [14]. The factor of the average mother’s income in a county was represented by the PCI in the corresponding county. Another vital indirect factor is the proportion of hospital births, which reflects the accessibility of hospitals and skilled midwives [12]. The third factor is antenatal care, which helps detect obstetric diseases so that pregnant women can receive timely treatment [14]. The determinant of the proportion of hospital births was quantified as the proportion of pregnant women who delivered in hospitals (PPWDH). The determinant of antenatal care was quantified as the proportion of pregnant women who received 5 or more maternal check-ups (PPWFC). The 2 covariates in a county were obtained by an average calculation for the county. The proxies are depicted in Fig 1. Data analyses were performed as per a prespecified protocol (S1 Text). This study followed the Strengthening the Guidelines for Accurate and Transparent Health Estimates Reporting (GATHER) Checklist (S1 GATHER Checklist).

### Data sources

**Fig 2:**
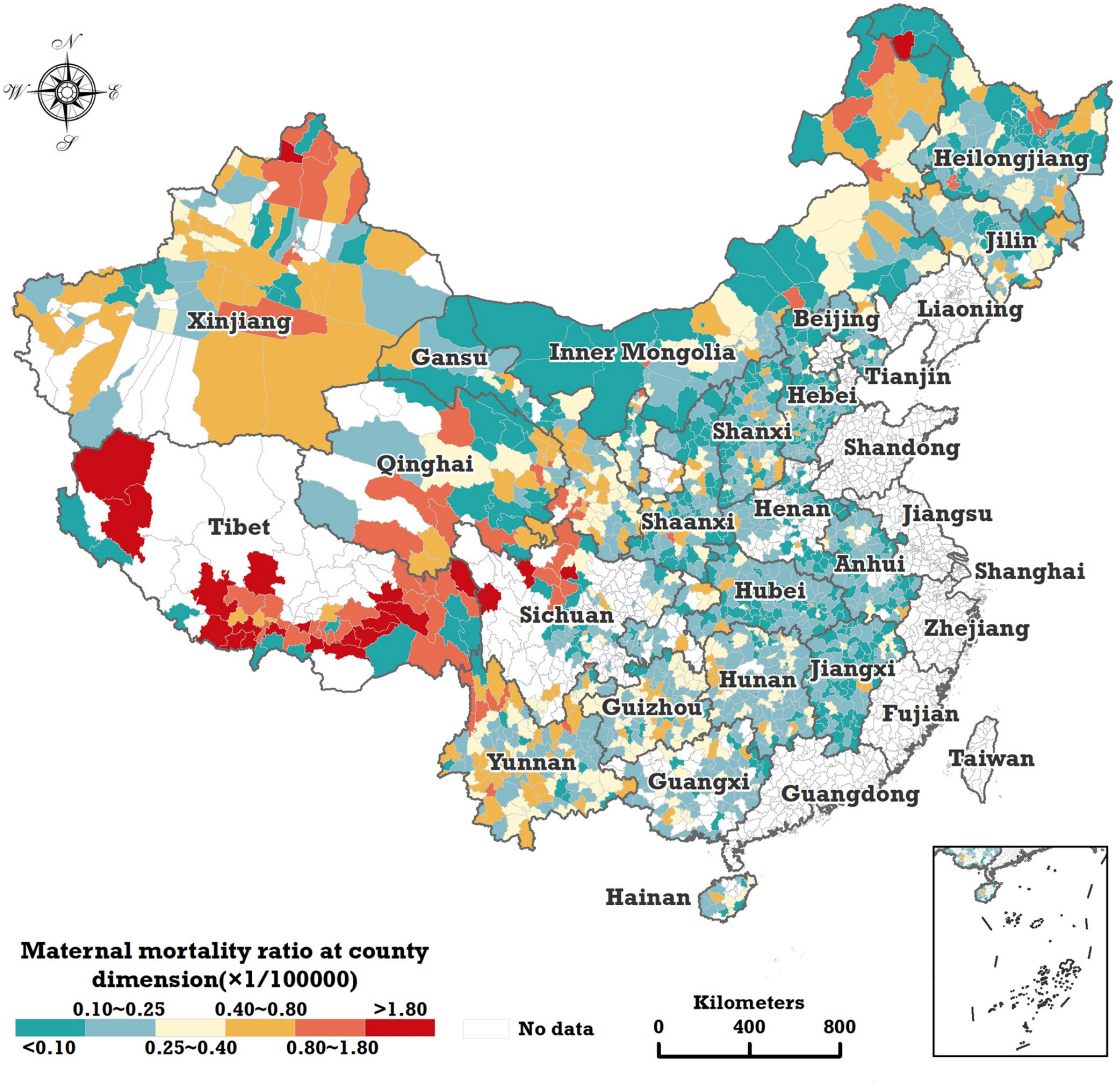
Spatial distribution of the mean MMR in 1,832 counties in China from 2010 to 2013. MMR, maternal mortality ratio.

We used county-level maternal mortality data from the National Maternal and Child Health Surveillance System (NMCHSS) over the 2010–2013 period, which covers 1,832 counties of China (Fig 2), including a total of 6,348 maternal death cases and about 33.74 million live births. The other counties, shown as blank areas in Fig 2, were not surveilled by the NMCHSS. The county-level data for PCI for the period 2010–2013 were collected from the China County Statistical Yearbook for the corresponding years. The raw data for the 2 other covariables, PPWDH and PPWFC, from 2010 to 2013 were collected from the NMCHSS. The geographical base map data, including provincial and prefectural boundaries, were collected from the Institute of Geographic Sciences and Natural Resources Research, Chinese Academy of Sciences (http://www.resdc.cn/).

### Statistical methods

#### Bayesian space–time model

To reveal the spatiotemporal pattern and influencing patterns of the MMR in China, we have applied a Bayesian space–time model [15] to the data from the participating counties. Considering the low probability of maternal mortality, to model the count data for rare events, we integrated the zero-inflation Poisson (ZIP) model [16–18] into the Bayesian space–time model. The number of maternal deaths in each county in every year follows a Poisson distribution, expressed by formulas 1–3:

$$y_{it}\sim Poisson(\mu_{it})$$ (1)

$$\mu_{it}=(1-u_{it})n_{it}\theta_{it}$$ (2)

$$u_{it}\sim Bern(p_{0})$$ (3)

*y*_*it*_ and *n*_*it*_ denote the numbers of maternal mortalities and live births, respectively, in a county *i* (*i* = 1, 2, …, 2,205) in a year *t* = (1, 2, 3, 4). *μ*_*it*_ is the average of the maternal death count, *u*_*it*_ is the parameter of ZIP, *θ*_*it*_ is the estimation of the MMR, *p*_0_ is the probability of zero maternal deaths. The spatiotemporal evolutionary progress of the MMR can be described as follows:

$$ln\left(\theta_{it}\right)=\alpha +s_{i}+(b_{0}t^{*}+v_{t})+b_{1i}t^{*}+\varepsilon_{it},$$ (4)

where *exp*(*s*_*i*_) represents the overall relative spatial risk of the MMR in the *i*-th county; *exp*(*s*_*i*_) directly quantifies that the MMR in the *i*-th county is *exp*(*s*_*i*_) times higher than the overall MMR over the study area if *exp*(*s*_*i*_) is greater than 1.0 and vice versa. t* = *t* − 2.5 represents the mid-observation period, and *α* is the overall logarithm of maternal mortality risk in China over the 4 years. The variable *b*_0_ represents the overall rate of change in the maternal mortality risk, and *v*_*t*_ signifies the additional Gaussian noise detecting nonlinear trend. (*b*_0_t* + *v*_*t*_) describes the global trend. The term *b*_1_t* describes the local trend of each county; *exp*(*b*_1*i*_) directly indicates that the local trend of the MMR in the *i*-th county is *exp*(*b*_1*i*_) times stronger than the global trend over the study period if *exp*(*b*_1*i*_) is greater than 1.0 and vice versa.*ε*_*it*_ represents a random effect.

Referring to Fig 1, we selected the 3 main covariates as follows: PCI in each county, PPWDH in each county, and PPWFC in each county. The influencing effects were estimated by the Bayesian multivariable regression model [19] as follows:

$$\theta_{i\_A}=s_{i}+\beta_{PCI}x_{i\_,PCI}+\beta_{PPWDH}x_{i\_,PPWDH}+\beta_{PPWFC}x_{i\_,PPWFC}+\varepsilon_{i\_},$$ (5)

$$\theta_{i\_A}=s_{z(i)}+\beta_{PCI(z(i))}x_{i\_,PCI}+\beta_{PPWDH(z(i))}x_{i\_,PPWDH}+\beta_{PPWFC(z(i))}x_{i\_,PPWFC}+\varepsilon_{i\_}.$$ (6)

*θ*_*i*_*A*_ represents the timely average MMR in the *i*-th county. The terms *x*_*i*_,*PCI*_, *x*_*i*_,*PPWDH*_, and *x*_*i*_,*PPWFC*_ are vectors of the 3 above-mentioned covariates and explain the variations in the timely average MMR in county *i*. The terms *β*_*PCI*_, *β*_*PPWDH*_, and *β*_*PPWFC*_ are the overall regression coefficients; *β*_*PCI*(*z*(*i*))_, *β*_*PPWDH*(*z*(*i*))_, and *β*_*PPWFC*(*z*(*i*))_ are the zonal regression coefficients; and *s*_*i*_ and *s*_*z*(*i*)_ are intercept terms. *ε*_*i*__ represents random effects.

The overall spatial random effect *s*_*i*_ is the conclusion of a spatially structured random effect and a spatially unstructured random effect; the latter follows a Gaussian distribution. To impose spatial structure, we used the prior conditional autoregressive (CAR) [20] value with an adjacency matrix *W* of size *N* × *N* (*N* is the number of counties), where its diagonal entries *w*_*ij*_ = 1 if areas *i* and *j* share a common boundary; otherwise, *w*_*ij*_ = 0. The posterior probability of the parameter *exp*(*s*_*i*_) being greater than 1.0, denoted as *p*(*exp*(*s*_*i*_) > 1.0|*data*), can be inferred through the Bayesian space–time model (formulas 1–4). We classified the counties by a 2-stage classification rule [21]. In the first stage, we defined a county as a hotspot if the posterior probability *p*(*exp*(*s*_*i*_) > 1 | data) was greater than 0.8 and as a coldspot if the posterior probability *p*(*exp*(*s*_*i*_) > 1 | data) was less than 0.2. We defined the other counties as neither hotspots nor coldspots, instead classifying them as warmspots. In the second stage, we further classified a county under each risk category in the first stage into one of three trend patterns based on the posterior probability of the parameter *exp*(*b*_1*i*_) being greater than 1.0, denoted as *p*(*exp*(*b*_1*i*_) > 1|*data*). That is, a country was classified as having a stronger local trend than the global trend if *p*(*exp*(*b*_1*i*_) > 1|*data*) ≥ 0.8, a weaker local trend than the global trend if *p*(*exp*(*b*_1*i*_) > 1|*data*) ≤ 0.2, and a local trend approximating the global trend if 0.2 < *p*(*exp*(*b*_1*i*_) > 1|*data*) < 0.8.

The Bayesian estimation employs WinBUGS 14 [22]. The number of iterations for each chain was set at 150,000, of which 100,000 were for the burn-in period and 50,000 were for the number of iterations of the posterior distribution of parameters. The convergence was evaluated with the Gelman–Rubin statistical parameter estimation, in which the closer the value is to 1, the better the convergence is. The Gelman–Rubin parameters [23] of all parameters in this study ranged from 0.99 to 1.01, indicating the steady convergence of these statistical results.

#### GeoDetector model

To further explore the patterns of influence of the MMR, especially the interaction of multiple factors, this study employed the GeoDetector model [24,25]. The axiom of GeoDetector is that 2 variables would be (linearly or nonlinearly) coupled in strata if one causes another. The GeoDetector can not only quantify the determinant power of a single factor to an independent variable, but it can also estimate the interactive effects of different factors. The GeoDetector model uses a *q*-statistic value to quantify the magnitude of the influencing power of the single factor or different interacted factors. The *q*-statistic value can be expressed as follows:

$$q=1-\frac{\sum_{h=1}^{l}N_{h}\sigma_{h}^{2}}{N\sigma^{2}}\times 100\%.$$ (7)

*h* (*h* = 1,2, …, *l*) represents the spatial stratification of the single factor *X* or the crossed strata of multifactor *X* values, e.g., *X*1 ∩ *X*2, *N*_*h*_ and *N* are the numbers of units in subregion *h* and the entire area, separately. $\sigma_{h}^{2}$ and *σ*^2^ are the variances of the MMR in subregion *h* and in the entire area, respectively. The *q*-statistic value quantifies the deterministic power of a single factor or of the interactive factors. The *q*-statistic value is between 0% and 100%, and the larger the *q*-statistic value, the larger the deterministic power of a single factor or 2 interactive factors on MMR. The interactive effect is nonlinearly enhanced if the interaction *q*-statistic value, denoted as *q*(*X*1 ∩ *X*2), is greater than the sum of the corresponding 2 single *q*-statistic values, denoted as *q*(*X*1) + *q*(*X*2); is enhanced if *q*(*X*1 ∩ *X*2) is greater than the maximum of *q*(*X*1) and *q*(*X*2); is independent if *q*(*X*1 ∩ *X*2) is equal to *q*(*X*1) + *q*(*X*2); and is weakened if *q*(*X*1 ∩ *X*2) is less than any of *q*(*X*1) and *q*(*X*2). The significance of the *q*-statistic value was conducted by F test [25], and the significance was set at 0.05.

## Results

### Spatiotemporal trends

**Fig 3:**
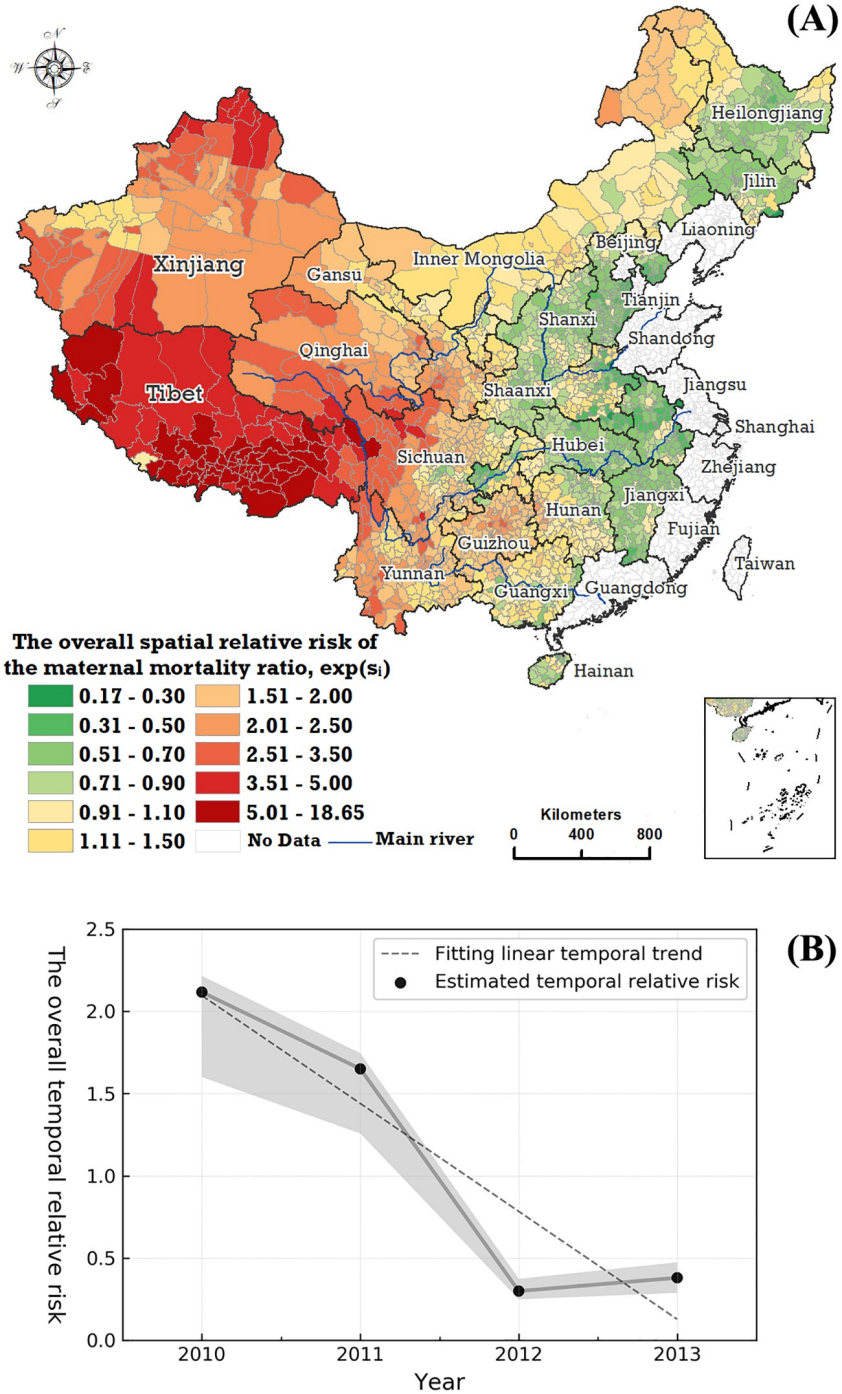
The overall spatial relative risk trends of the MMR in China over the period 2010–2013. (A) The posterior median of the spatial relative risks (exp(*s*_*i*_)) in counties and (B) the overall temporal trend, with the posterior median of the temporal relative risk (*exp*(*b*_0_t* + *v*_*t*_)) having a probability of 95%. MMR, maternal mortality ratio.

Generally, the spatial distribution of the mean MMR in 1,832 counties in China from 2010 to 2013 (Fig 2) shows that the 3 western provincial regions—Tibet, Qinghai, and Xinjiang—experienced the highest MMR, and the counties with the highest level of MMR with greater than 1.80 per 100,000 live births were mainly located in Tibet and Western Sichuan. Fig 3 shows the estimated overall spatial and temporal trends of the MMR from the Bayesian space–time model mentioned above. Provincial areas where the surveillance data were fully not available are developed coastal regions, where the MMR is very low.

Fig 3A shows a common spatial pattern structure of the MMR in the Chinese mainland over the period 2010–2013. From east to west, the 2,205 counties can be separated into 3 areas. In Fig 3A, the counties shaded in green are located near the east coast and have a low level of common relative risk (<1.0) in the MMR. In other words, these counties experience a relatively lower MMR. The counties located in Tibet, Xinjiang, Western Sichuan, and Qinghai in western China have a higher level of common spatial relative risk (>2.5), with the level in most of the monitored counties in Tibet estimated at more than 3.5. Specifically, the western and southwestern counties shaded in deep yellow or red in Fig 3A have a relatively high MMR compared with the overall level in the country. The counties located in the northeastern, north-central, and southwestern regions have an average MMR level. The results suggest that the spatial distribution of the MMR in China has formed an explicit spatial gradient structure. The overall temporal relative risk can be estimated by use of the Bayesian space–time model. The results show that the overall temporal trend of the MMR presented a general decrease from 2010 to 2013 (Fig 3B). Furthermore, a sharp decline occurred from 2011 to 2012, followed by a slight increase from 2012 to 2013.

We classified the 2,205 counties into 3 categories—hotspots, coldspots, and warmspots—based on the 2-stage classification rules, as previously outlined. Among the 2,205 counties, 925 (42.0%) are classified as hotspot regions and 764 (34.6%) as coldspot regions; 516 (23.4%) counties are identified as warmspot regions. Two contiguous areas are identified as hotspots, shaded in red in S1 Fig. The smaller of the hotspot areas is located in Inner Mongolia and the north of Heilongjiang Province, while the larger area covers almost all of western and southwestern China.

**Fig 4:**
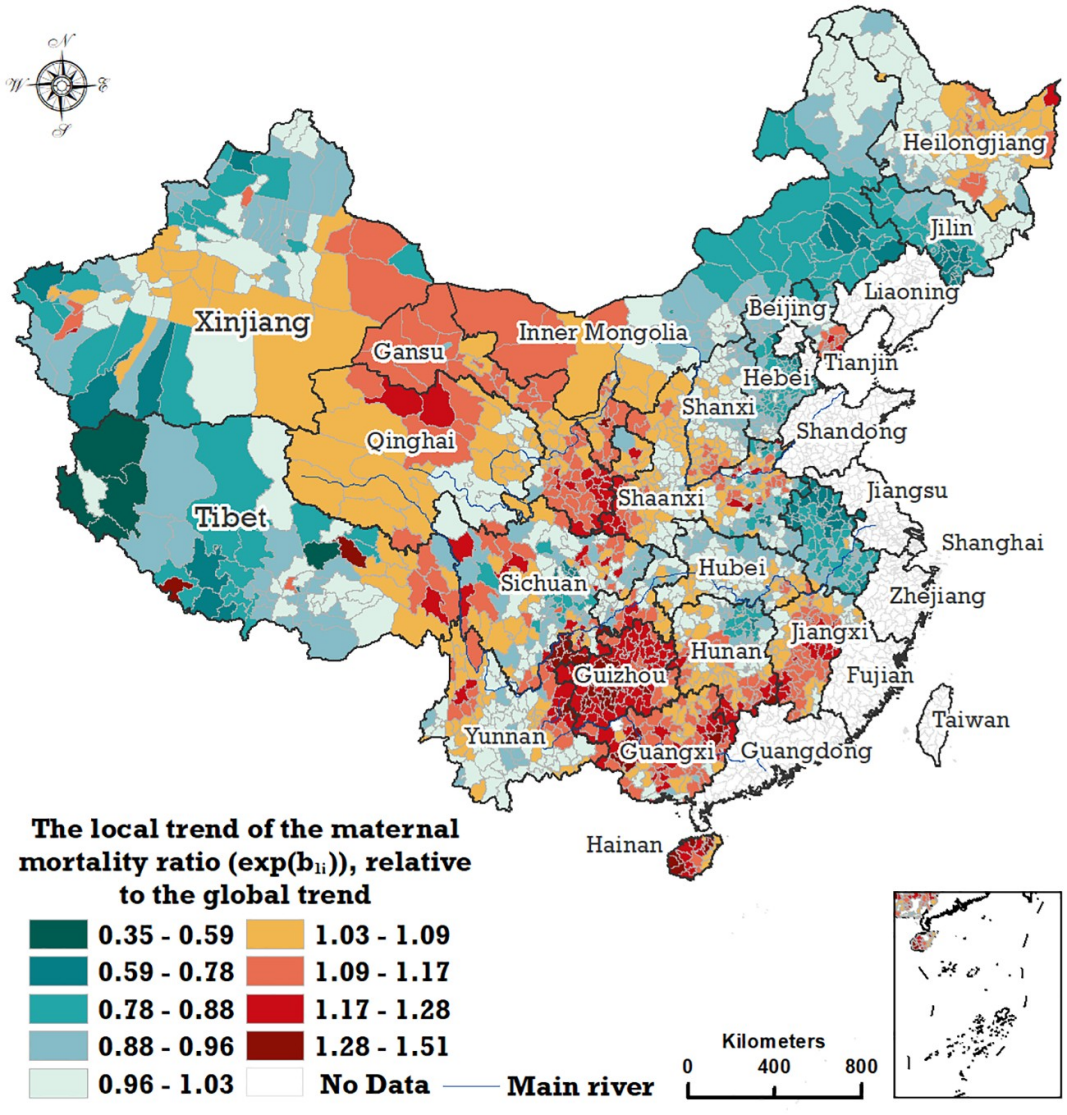
The local trend of the MMR in 2,205 counties, relative to the global trend, with the posterior medians of the parameter *exp*(*b*_1i_). MMR, maternal mortality ratio.

The local trend, quantified by the parameter *exp*(*b*_1*i*_), can be estimated through the Bayesian space–time model. Considering that the overall temporal trend exhibited a downward movement, the local trend of the MMR in the *i*-th county has a stronger downward trend than the overall downward trend if the parameter *exp*(*b*_1*i*_) is greater than 1.0 and has a weaker downward trend than the overall downward trend if *exp*(*b*_1*i*_) is less than 1.0. As illustrated in Fig 4, the spatial distribution of the local temporal trend shows that there is a continuous zone from the northwest to the southeast that has a stronger local downward trend than the national downward trend, with Heilongjiang’s northern and eastern regions also experiencing a stronger local downward trend. Most of the areas of Tibet and west Xinjiang and the areas near the eastern regions of China indicate a weaker decline in the local trend than the national downward trend.

Table 1 lists the cross-classified results with the 2-stage classification rule. Of the 925 hotspot counties, most (552 counties, accounting for 59.7%) experienced a stronger local downward trend in the MMR, while the rest (332 counties, accounting for 35.9%) experienced a weaker local downward trend. 223 counties, accounting for 29.2% of the 764 coldspot counties, experienced a weaker local downward trend in the MMR, while the local trends in the MMR of 475 coldspot counties, accounting for 62.2% of coldspot counties, approximated to the national downward trend. Of the 516 warmspot counties, 100 (accounting for 19.4%) experienced a stronger local downward trend than the national downward trend, while 77 (accounting for 14.9%) experienced a weaker trend, and 339 warmspot counties, accounting for 65.7%, approximated to the national downward trend.

**Table 1:** Number of counties resulting from the cross classification (2,205 counties).

	Stronger Decreasing Local Trend	Weaker Decreasing Local Trend	Approximate to the National Decreasing Trend	Total
**Hotspots**	552 (59.7%)	332 (35.9%)	41 (4.4%)	925 (100.0%)
**Coldspots**	66 (8.6%)	223 (29.2%)	475 (62.2%)	764 (100.0%)
**Warmspots**	100 (19.4%)	77 (14.9%)	339 (65.7%)	516 (100.0%)

### Patterns of influence

#### Bayesian estimates

As has been noted, the explanatory variables include PCI (unit: thousands of Chinese Yuan), PPWDH (%), and PPWFC (%). To detect whether the variables are closely correlated, we calculated Pearson’s correlation coefficients between them. The statistical test probability values of all Pearson’s correlation coefficients are all less than 0.001 (*p* < 0.001). The correlation coefficients between PCI and PPWDH, PCI and PPWFC, and PPWDH and PPWFC were 0.19, 0.14, and 0.52, respectively; this suggests that the 3 variables are not strongly correlated. According to Pearson’s correlation coefficients of the 3 variables PCI, PPWDH, and PPWFC, we incorporated them to explain the determinants of the MMR using the Bayesian multivariable regression. We have estimated the overall national regression parameters and subnational regression parameters of the 3 variables for China’s eastern and southern coastlands, central and northern regions, and western and southwestern regions (S2 Fig). Notably, although the 2,205 counties are mostly located in western or central China, 306 of the counties are located in Hebei, Guangxi, and Hainan Provinces, in China’s eastern and southern coastlands. We then investigated the regional influencing mechanism for the 3 subnational regions.

The national and subnational regression parameters can be estimated from formulas 5 and 6. Table 2 lists the estimated Bayesian regression results of the nation and of 3 subnational regions: the eastern and southern coastlands, central and northern regions, and western and southwestern regions (S2 Fig). Nationally, there is a significant negative relationship between the MMR and the factors of PPWDH and PPWFC; PCI, however, is not significantly related to the MMR. Furthermore, PPWDH is the major influencing factor nationwide, while PPWFC is the second major influencing factor. The MMR decreased by 1.787 (95% confidence interval [CI] 1.424–2.142, *p* < 0.001) per 100,000 live births when PPWDH increased by one percentage point if PCI and PPWFC were invariant. The MMR decreased by 0.623 (95% CI 0.436 to 0.798, *p*<0.001) per 100,000 live births when PPWFC increased by one percentage point if PCI and PPWDH were invariant.

**Table 2:** Overall regression parameters and subnational regression parameters of the 3 variables in China’s eastern and southern coastlands, central and northern regions, and western and southwestern regions, with a 95% CI, estimated by posterior means of the Bayesian multivariable regression model.

Group	PCI	PPWDH	PPWFC
**Nationwide**	−0.163 (−0.967, 0.656); $P_{\beta_{PCI\langle 0|data}}=65.0\%$	−1.787 (−2.142, −1.424); $P_{\beta_{PPWDH\langle 0|data}}=99.9\%$	−0.623 (−0.798, −0.436); $P_{\beta_{PPWFC\langle 0|data}}=99.9\%$
**Eastern and southern coastlands**	−0.723 (−1.279, −0.195); $P_{\beta_{PCI\langle 0|data}}=99.7\%$	0.195 (−0.581, 1.017); $P_{\beta_{PPWDH\langle 0|data}}=33.2\%$	0.058 (−0.182, 0.276); $P_{\beta_{PPWFC\langle 0|data}}=31.5\%$.
**Central and northern regions**	0.177 (−0.281, 0.677); $P_{\beta_{PCI\langle 0|data}}=23.8\%$	0.223 (−0.473, 0.905); $P_{\beta_{PPWDH\langle 0|data}}=25.3\%$	0.042 (−0.076, 0.152); $P_{\beta_{PPWFC\langle 0|data}}=23.7\%$
**Western and southwestern regions**	−1.111 (−3.665, 1.485); $P_{\beta_{PCI\langle 0|data}}=80.0\%$	−0.081 (−0.808, 0.660); $P_{\beta_{PPWDH\langle 0|data}}=58.6\%$	−1.686 (−2.090, −1.275); $P_{\beta_{PPWFC\langle 0|data}}=99.9\%$

**Abbreviations**: CI, confidence interval; PCI, per capita income; PPWDH, proportion of pregnant women delivering in hospitals; PPWFC, proportion of pregnant women who had at least 5 check-ups.

Different patterns of influence are exhibited in the 3 regions of China, which include the eastern and southern coastlands, the central and northern regions, and the western and southwestern regions. The results (Table 2) show that PCI is the significant influencing factor in the eastern and southern coastlands of China, while PCI and PPWFC are the significant influencing factors in the western and southwestern regions of China; there is no significant influencing factor in the central and northern regions of China. In China’s eastern and southern coastlands, the MMR decreased by 0.723 (95% CI 0.195–1.279, *p* = 0.003) per 100,000 live births when PCI increases by 1,000 Chinese Yuan if PPWDH and PPWFC were invariant. In the western and southwestern regions, where the major influencing factor is PPWFC, the MMR decreased by 1.686 (95% CI 1.275–1.275, *p* < 0.001) per 100,000 live births when PPWFC increased by one percentage point if PCI and PPWDH were invariant. The MMR decreased nonsignificantly by 1.111 (95% CI −1.485 to 3.665, *p* < 0.20) per 100,000 live births when PCI increases by 1,000 Chinese Yuan if PPWDH and PPWFC were invariant.

#### GeoDetector statistical results

In terms of the univariate, the GeoDetector results (Table 3) are consistent with the above estimated Bayesian results. Nationwide, PPWDH and PPWFC are the major influencing factors, and their influence on the MMR is 24.1% (*p* < 0.001) and 21.5% (*p* < 0.001), respectively, while the influence of PCI is only 2.2% (*p* < 0.001). In the eastern and southern coastlands of China, PCI’s influence (9.2% [*p* = 0.024]) is greater than that of the other 2 factors of PPWDH (4.2% [*p* = 0.895]) and PPWFC (2.7% [*p* = 0.950]). Analogous with the Bayesian statistical results, the 3 factors’ GeoDetector *q*-statistic values for the central and northern regions of China are all less than 3.0%. Moreover, the GeoDetector results show that PPWFC and PPWDH are the important influencing factors in the western and southwestern regions of China, as their influences are 29.3% (*p* < 0.001) and 26.0% (*p* < 0.001), respectively, while the influence of PCI is only 2.5% (*p* = 0.472). This is inconsistent with the Bayesian results.

**Table 3:** GeoDetector *q*-statistic value (F test value) for the influencing power of a single factor at the county level in China’s eastern and southern coastlands, central and northern regions, and western and southwestern regions.

Group	PCI	PPWDH	PPWFC
**Nationwide**	2.2% (*p* < 0.001)	24.1% (*p* < 0.001)	21.6% (*p* < 0.001)
**Eastern and southern coastlands**	9.2% (*p* = 0.024)	4.2% (*p* = 0.895)	2.7% (*p* = 0.950)
**Central and northern regions**	2.6% (*p* = 0.192)	1.0% (*p* = 0.951)	0.9% (*p* = 0.946)
**Western and southwestern regions**	2.5% (*p* = 0.472)	26.0% (*p* < 0.001)	29.3% (*p* < 0.001)

**Abbreviations**: PCI, per capita income; PPWDH, proportion of pregnant women delivering in hospitals; PPWFC, proportion of pregnant women who had at least 5 check-ups.

Table 4 presents the GeoDetector statistical results for interacting factors at the national and subnational levels. The results suggest that the nonlinear enhanced interactive effect occurred not only at the national level but also at the 3-regional level (the eastern and southern coastlands, central and northern region, and western and southwestern region). In this regard, the influencing power of the interacting factors of PPWDH and PPWFC on the national level is up to 47.2% (*p* < 0.001). In combination with the Bayesian estimated results, this indicates that the integrated implementation of medical intervention or assistance, delivery in hospital (the proxy variable PPWDH), and regular maternal check-ups (the proxy variable PPWFC) can efficiently reduce the MMR. At the same time, the interactive influencing power of PCI and PPWDH (41.7% [*p* < 0.001]) is more than that of PCI and PPWFC (34.4% [*p* < 0.001]). The above shows the pattern of interactive influence on the national scale. For the 3 subnational regions, the greatest interactive influencing effect is in the western and southwestern regions, while the weakest is that in the central and northern regions. Furthermore, although the individual influencing power of the 3 factors in the eastern and southern coastlands are all less than 10.0%, the interactive influencing power of the 3 factors in that region is markedly higher. The minimum is 17.3% (*p* < 0.001) (PPWDH and PPWFC), and the maximum is up to 35.1% (*p* < 0.001) (PCI and PPWFC), implying that the joint influences of the 3 factors, especially PCI and the other 2 factors, have a significant impact on the MMR in the eastern and southern coastlands. The strongest interactive influencing effect occurs in the western and southwestern regions of China, where the GeoDetector *q*-statistics values all exceed 80.0%. Nonetheless, the influencing power of the single factor of PCI is only 2.5% (*p* = 0.472) in the western and southwestern regions, while the interactive influencing power of PCI and the other 2 factors, PPWDH and PPWFC, are 82.5% (*p* < 0.001) and 80.1% (*p* < 0.001), respectively. The other interactive influencing power, between PPWDH and PPWFC, is 82.7% (*p* < 0.001). The results suggest that the 3 factors PCI, PPWDH, and PPWFC can be regarded as the major influencing factors in the western and southwestern regions, i.e., they may account for more than 80% of the variability in the MMR in the western and southwestern regions of China. In the central and northern regions, despite an increase, the interactive influencing power of the 3 factors is still less than 20.0%; this again indicates that the 3 factors are not yet the major influencing factors in the MMR of these regions.

**Table 4:** GeoDetector *q*-statistics value (F test value) for the influencing power of interacting factors at the county level in China’s eastern and southern coastlands, central and northern regions, and western and southwestern regions.

Group	PCI ∩ PPWDH	PCI ∩ PPWFC	PPWDH ∩ PPWFC
**Nationwide**	41.7% (*p* < 0.001)	34.4% (*p* < 0.001)	47.2% (*p* < 0.001)
**Eastern and southern coastlands**	21.0% (*p* < 0.001)	35.1% (*p* < 0.001)	17.3% (*p* < 0.001)
**Central and northern regions**	10.5% (*p* < 0.001)	18.2% (*p* < 0.001)	8.5% (*p* = 0.281)
**Western and southwestern regions**	82.5% (*p* < 0.001)	80.1% (*p* < 0.001)	82.7% (*p* < 0.001)

**Abbreviations**: PCI, per capita income; PPWDH, proportion of pregnant women who delivered in hospitals; PPWFC, proportion of pregnant women who had at least 5 check-ups.

## Discussion

In this study, we used a Bayesian space–time model integrated with the ZIP model to explore spatiotemporal trends in the MMR of 2,205 Chinese counties from 2010 to 2013. We found that, although China has decreased the MMR in recent decades, temporal and spatial heterogeneity still exists. The different patterns of influence of the 3 main ecological determinants of the MMR at national and subnational level were identified through the utilisation of a Bayesian multivariable regression model and GeoDetector *q*-statistics model. Nationally, medical intervention, PPWDH, and PPWFC are the major determinants. The major influencing factors in China’s western and southwestern regions are PCI and PPWFC, while in China’s eastern and southern coastlands, it is PCI. Moreover, China’s western and southwestern regions demonstrated the strongest impacts from the interaction of the different factors.

Previous research conducted by Liang and colleagues [1] has estimated the MMR in 2,852 Chinese counties based predominantly on data sourced from the national Annual Report System on Maternal and Child Health (ARMCH), whose source’s data under-reported maternal deaths, and calculated the annual rate of decline for the MMR without considering spatiotemporal correlation. Our study uses the data from the NMCHSS with more accurate MMR data to identify the temporal and spatial trends from the complicated spatiotemporal coupling process of the MMR in China. Additionally, our study also estimates the main ecological determinants for the MMR at the national and subnational levels in China. The Bayesian estimated overall spatial trends for the period 2010–2013 in our study are generally similar to, though not exactly the same as, the spatial patterns in the MMR for China in 2015 that are demonstrated in Liang and colleagues’ study [1]. Specifically, a distinct gradient structure with a gradually higher MMR from the east to the west has been stable. Three provincial areas—Tibet, Xinjiang, and Qinghai—showed the highest level of MMR in China. Five provincial areas—Inner Mongolia, Ningxia, Sichuan, Guizhou, and Yunnan—demonstrated the second-highest level of MMR. Furthermore, the MMR in most of western and southwestern China is at a higher level than the national average, with the hotspots classified by the posterior probability of the spatial relative risk being greater than 1.0. Different local trends in the MMR appeared throughout China. Liang and colleagues [1] also calculated the annual average rate of decrease in maternal mortality at the county level in mainland China from 1996 to 2015. However, the spatial patterns of local trends estimated in our study differ from those of Liang and colleagues. The local trends estimated by our study, decomposed from the overall trend and considering the spatiotemporal correlation, possess a more striking spatial structure than those calculated by Liang and colleagues’ study, which were calculated independently for each county. We argue that the local trends with distinct spatial structure may have a closer relation to reality and therefore provide a more significant reference for the formulation of public health policies. It should be pointed out that the local trends in the MMR in the northwestern and southwestern regions and eastern Heilongjiang exhibited a stronger downward trend than the national average downward trend and that most of these regions simultaneously have a higher MMR and a stronger downward trend simultaneously. In addition, this study also notes that while the counties in the western provinces of Tibet, Qinghai, and Xinjiang have the highest level of the MMR, they have a weaker downward trend than the national downward trend (Fig 4).

A major strength of our study is its use of the state-of-the-art Bayesian space–time model, which can disassemble the overall spatial relative risk, overall temporal trend, and local trend from the complex space–time coupling process to closely investigate the spatiotemporal heterogeneities in the MMR in Chinese 2,205 counties. Another strength of this research is its estimation of the quantitative influencing effects of the 3 main determinants—PCI, PPWDH, and PPWFC—at the national and subnational levels through the use of the Bayesian multivariable regression model. The explanatory power of a single factor in the MMR and the interactive explanatory power of any two of the three factors were investigated using the GeoDetector model. A better understanding of the quantified ecological determinants of the MMR would support evidence-based policymaking to sustainably reduce the MMR in China and other developing areas worldwide.

Our study has several limitations. First, the registration system does not cover the entire country because the eastern coastal counties have very low maternal mortality, and therefore, the results do not represent the total trend of maternal mortality throughout China. Second, we only have the monitoring data for the years 2010–2013, a limited time period. Although the spatiotemporal trends may remain relatively steady, the space–time variability of recent years should be investigated. Third, the influencing factors discussed in this research could be more comprehensive; besides the 3 factors of PCI, PPWDH, and PPWFC, there are certainly other influencing factors that should form the focus of future research, such as accessibility of blood banks, road conditions, and the natural environment.

There are currently still many counties in China with an MMR higher than the SDGs target; appropriate interventions at the national and subnational level should be devised to reduce avoidable maternal mortality. An in-depth study of patterns of influence in necessary to inform effective interventionist policymaking. Our study investigated the influencing factors of maternal mortality in China through 2 approaches; the results suggest that nationally, medical intervention factors, PPWDH, and PPWFC are major influencing factors. Social and economic factors are not the principal factors at the national level but are major influencing factors in the western and southwestern regions. Interestingly, the strength of influence of PPWDH nationwide is greater than that it is in the western and southwestern regions, and the strength of influence of PPWFC nationwide is less than it is in the western and southwestern regions. This indicates that antenatal care should be strongly reinforced in the western and southwestern regions, while nationwide, the percentage of hospital births should first be improved and the number of midwives increased, at which point antenatal care should also be improved. It is also important to raise the income levels of birth families in the western and southwestern regions and eastern and southern coastlands. Public medical resources and conditions are better in the eastern and southern coastlands, and therefore, the social and economic factors have a major influence on the MMR in these areas. Additionally, this study did not find a significant influencing factor for the maternal mortality in central and northern regions, where socioeconomic development and public medical conditions are at an intermediate level compared to the national standard [26].

Despite the generally decrease of the MMR, it nonetheless presents a highly spatial heterogeneity. Some underdeveloped areas from the western and southwestern regions not only have a higher MMR but also show a weaker downward trend. Traditional customs, poor (health) education, and inadequate medical resources are 3 major determinants of the MMR in these areas. The issue of traditional customs is linked with the proportion of minority or indigenous population. According to the Chinese Population Census data for 2010, the 4 western provincial regions with a higher MMR—Tibet, Xinjiang, Qinghai, and Yunnan—also had a higher proportion of minority population—91.8%, 59.5%, 47.0%, and 33.4%, respectively. However, the proportion of the minority or indigenous population for 4 central and northern provincial regions that were coldspot areas for the MMR—Shanxi, Hubei, Jiangxi, and Jilin—were 0.3%, 4.3%, 0.3%, and 8.0%, respectively (http://www.stats.gov.cn/tjsj/pcsj/rkpc/6rp/indexch.htm). The proportion of women whose highest educational level was elementary school or below in the 4 western provincial regions of Tibet, Xinjiang, Qinghai, and Yunnan was 83.5%, 37.1%, 52.9%, and 53.9%, respectively, which is higher than that of other provincial regions (http://www.stats.gov.cn/tjsj/ndsj/2014/indexch.htm). Influenced by traditional customs, women in these areas, where the proportion of the ethnic minority or indigenous population is high, prefer to give birth at home [27–30]. Low levels of education or even illiteracy may limit the ability or willingness of women to seek the provision of formal healthcare services [31]. Excluding the factors of the proportion of ethnic minorities in the population and the educational level, the maternal and healthcare medical resources in China’s western and southwestern regions are also at a disadvantage. According to the China Health and Family Planning Statistical Yearbook for 2014 (http://tongji.cnki.net/kns55/navi/YearBook.aspx?id=N2014120147&floor=1), the number of medical personnel in maternal and child healthcare service institutions per 1,000 persons for the 4 western provincial regions was 0.13, 0.15, 0.10, and 0.13, respectively. These figures were all lower than those for the 4 central and northern regions of Shanxi, Hubei, Jiangxi, and Jilin, which were 0.16, 0.23, 0.23, and 0.16, respectively. To sum up, in the 3 aspects of traditional customs, the education level of female residents, and maternal and healthcare medical resources, the western and southwestern regions were all disadvantaged. Although the factors of the traditional beliefs and the low education level of female residents cannot be rapidly addressed, the strengthening of the provision of health education and care to pregnant women can be implemented in the areas with the highest MMR risk.

The hospital delivery and prenatal examination rates in the western provinces were far lower than the national average; the average rates of hospital delivery and receipt of 5 or more maternal check-ups in Tibet were only 62.5% and 25.7%, respectively, while for Qinghai, this was 90.4% and 59.6%. The rural areas where hospital delivery rates are less than 50% were located mainly in Tibet, Sichuan, and Qinghai, while the rates of hospital delivery and receipt of 5 or more maternal check-ups were 99.6% and 91.1% in Hebei, 99.4% and 83.4% in Shanxi, and 99.6% and 90.0% in Jilin, respectively. This indicates that there is also much room for improvement in the provision of healthcare for pregnant women in the western regions. As mentioned above, because of a lack of education, many women in the western regions do not know the importance of prenatal care and rarely go to hospitals to receive it. Income growth also plays an important role; it is apparent that people with higher incomes have an advantage in accessing medical facilities and pay more attention to nutrition and health knowledge. Therefore, it is important that the government provides financial assistance to poor pregnant women and improves (health) education.

All in all, the income level of the birth family and medical intervention are the most important influencing factors, and this is further evident when considering the effect of the interaction of different factors. The results of this study suggest that intervention should focus on low-income families. Furthermore, this study provides some quantisation of influencing effects. Certainly, on account of the differing patterns of influence at the national and subnational levels, the corresponding diverse policies should be carefully devised to reduce the national and regional MMR. For those western and southwestern regions with a higher MMR risk, on the one hand, the health awareness of pregnant women should be raised through professional health education, while on the other hand, the rates of hospital delivery and receipt of 5 or more maternal check-ups should be increased by improving the conditions of county-level and township hospitals with better accessibility. Although China has achieved the SDGs target at the national level, 191 counties, mostly located in the western regions, had not reached the SDGs target in 2015 [1]. Further accurate and appropriate interventions need to be devised for these counties. The quantitative statistical results produced by this study can help the government to anticipate the effects of policy. While it is relatively difficult to increase the incomes of residents in the short term, a strengthening of public health interventions, such as the enhancement of healthcare provision for pregnant women, can be implemented relatively quickly.

A distinct gradient structure with a gradually higher MMR from the east to the west has been stable. An almost continuous zone from the northwest to the southwest experienced a strong downward trend in the MMR. This study identified 925 (hotspot) high-risk counties, mostly located in the western and southwestern regions, and 332 counties among them that are experiencing a slower downward trend than the overall national downward trend. The counties in the western provinces of Tibet, Qinghai, and Xinjiang have the highest level of MMR and a weaker downward trend than the national downward trend in these regions. Nationally, medical intervention, hospital delivery, and antenatal care are the major determinants. The major ecological determinants for the MMR in western and southwestern regions, which are developing areas, are PCI and PPWFC, while for developed areas, it is PCI. The interactive effects of different factors in China’s western and southwestern regions was the strongest, and the corresponding interactive influencing power of any two of the three factors PCI, PPWDH, and PPWFC were all greater than 80%.

## Supporting information

S1 Text Study protocol. (DOCX)

S1 GATHER Checklist GATHER, Guidelines for Accurate and Transparent Health Estimates Reporting. (DOCX)

S1 Fig Spatial distribution of the posterior probability of the overall spatial relative risk of the MMR, *P*(*exp*(*s*_*i*_)) > 1|*data*). MMR, maternal mortality ratio.
(TIF)

S2 Fig Illustration of China’s 3 regions—Eastern and southern coastland, central and northern regions, and western and southwestern regions. (TIF)

## Abbreviations

ARMCH: Annual Report System on Maternal and Child Health
CAR: conditional autoregressive
CI: confidence interval
GATHER: Strengthening the Guidelines for Accurate and Transparent Health Estimates Reporting
MDG: Millennium Development Goal
MMR: maternal mortality ratio
NMCHSS: National Maternal and Child Health Surveillance System
PCI: per capita income
PPWDH: proportion of pregnant women who delivered in hospitals
PPWFC: proportion of pregnant women who had at least 5 check-ups
SDG: Sustainable Development Goal
ZIP: zero-inflation Poisson

## References

[1] Liang J, Li X, Kang C, Wang Y, Kulikoff XR, Coates MM, et al Maternal mortality ratios in 2852 Chinese counties, 1996–2015, and achievement of Millennium Development Goal 5 in China: a subnational analysis of the Global Burden of Disease Study 2016. Lancet. 2019;393(10168):241–52. 10.1016/S0140-6736(18)31712-4 30554785 PMC6336935

[2] Alkema L, Chou D, Hogan D, Zhang S, Moller A-B, Gemmill A, et al Global, regional, and national levels and trends in maternal mortality between 1990 and 2015, with scenario-based projections to 2030: a systematic analysis by the UN Maternal Mortality Estimation Inter-Agency Group. Lancet. 2016;387(10017):462–74. 10.1016/S0140-6736(15)00838-7 26584737 PMC5515236

[3] Liang J, Dai L, Zhu J, Li X, Zeng W, Wang H, et al Preventable maternal mortality: geographic/rural-urban differences and associated factors from the population-based Maternal Mortality Surveillance System in China. BMC Public Health. 2011;11(1):243. 21501529 10.1186/1471-2458-11-243 PMC3108316

[4] Hogan MC, Foreman KJ, Naghavi M, Ahn SY, Wang M, Makela SM, et al Maternal mortality for 181 countries, 1980–2008: a systematic analysis of progress towards Millennium Development Goal 5. Lancet. 2010;375(9726):1609–23. 10.1016/S0140-6736(10)60518-1 20382417

[5] Say L, Chou D, Gemmill A, Tunçalp Ö, Moller A-B, Daniels J, et al Global causes of maternal death: a WHO systematic analysis. Lancet Global Health. 2014;2(6):e323–e33. 10.1016/S2214-109X(14)70227-X 25103301

[6] Kassebaum NJ, Bertozzi-Villa A, Coggeshall MS, Shackelford KA, Steiner C, Heuton KR, et al Global, regional, and national levels and causes of maternal mortality during 1990–2013: a systematic analysis for the Global Burden of Disease Study 2013. The Lancet. 2014;384(9947):980–1004. 10.1016/S0140-6736(14)60696-6 PMC4255481 24797575

[7] United Nations Development Programme (UNDP). UN hails China’s progress in achieving Millennium Development Goals in Final Report Jul 24, 2015. Internet [cited 2020 Jan 1]. https://www.cn.undp.org/content/china/en/home/presscenter/pressreleases/2015/07/-united-nations-hails-chinas-progress-towards-the-millennium-dev.html.

[8] Gao Y, Zhou H, Singh NS, Powell-Jackson T, Nash S, Yang M, et al Progress and challenges in maternal health in western China: a Countdown to 2015 national case study. Lancet Global Health. 2017;5(5):e523–e36. 10.1016/S2214-109X(17)30100-6 28341117 PMC5387688

[9] Du Q, Nass O, Bergsjo P, Kumar BN. Determinants for high maternal mortality in multiethnic populations in western China. Health Care for Women International. 2009;30(11):957–70. 10.1080/07399330903052137 19809900

[10] Li Q, Fottler M. Determinants of maternal mortality in rural China. Health Services Management Research. 1996;9(1):45–54. 10.1177/095148489600900105 10157222

[11] Khan KS, Wojdyla D, Say L, Gülmezoglu AM, Van Look PF. WHO analysis of causes of maternal death: a systematic review. Lancet. 2006;367(9516):1066–74. 10.1016/S0140-6736(06)68397-9 16581405

[12] Graham WJ, Witter S. Counting what counts for maternal mortality. Lancet. 2014;384(9947):933–5. 10.1016/S0140-6736(14)61604-4 25220960

[13] El Arifeen S, Hill K, Ahsan KZ, Jamil K, Nahar Q, Streatfield PK. Maternal mortality in Bangladesh: a Countdown to 2015 country case study. Lancet. 2014;384(9951):1366–74. 10.1016/S0140-6736(14)60955-7 24990814

[14] Chowdhury ME, Botlero R, Koblinsky M, Saha SK, Dieltiens G, Ronsmans C. Determinants of reduction in maternal mortality in Matlab, Bangladesh: a 30-year cohort study. Lancet. 2007;370(9595):1320–8. 10.1016/S0140-6736(07)61573-6 17933646

[15] Li G, Haining R, Richardson S, Best N. Space–time variability in burglary risk: A Bayesian spatio-temporal modelling approach. Spatial Statistics. 2014;9:180–91.

[16] Böhning D, Dietz E, Schlattmann P, Mendonca L, Kirchner U. The zero-inflated Poisson model and the decayed, missing and filled teeth index in dental epidemiology. Journal of the Royal Statistical Society: Series A (Statistics in Society). 1999;162(2):195–209.

[17] Hall DB. Zero-inflated Poisson and binomial regression with random effects: a case study. Biometrics. 2000;56(4):1030–9. 10.1111/j.0006-341x.2000.01030.x 11129458

[18] Xie M, He B, Goh T. Zero-inflated Poisson model in statistical process control. Computational Statistics & Data Analysis. 2001;38(2):191–201.

[19] Geisser S. Bayesian estimation in multivariate analysis. Annals of Mathematical Statistics. 1965;36(1):150–9.

[20] Besag J, York J, Mollié A. Bayesian image restoration, with two applications in spatial statistics. Annals of the Institute of Statistical Mathematics. 1991;43(1):1–20.

[21] Richardson S, Thomson A, Best N, Elliott P. Interpreting posterior relative risk estimates in disease-mapping studies. Environmental Health Perspectives. 2004;112(9):1016–25. 10.1289/ehp.6740 15198922 PMC1247195

[22] Lunn DJ, Thomas A, Best N, Spiegelhalter D. WinBUGS-a Bayesian modelling framework: concepts, structure, and extensibility. Statistics and Computing. 2000;10(4):325–37.

[23] Gelman A, Rubin DB. Inference from iterative simulation using multiple sequences. Statistical Science. 1992;7(4):457–72.

[24] Wang JF, Li XH, Christakos G, Liao YL, Zhang T, Gu X, et al Geographical detectors-based health risk assessment and its application in the neural tube defects study of the Heshun Region, China. International Journal of Geographical Information Science. 2010;24(1):107–27.

[25] Wang J-F, Zhang T-L, Fu B-J. A measure of spatial stratified heterogeneity. Ecological Indicators. 2016;67:250–6.

[26] Li Y, Wei YD. The spatial-temporal hierarchy of regional inequality of China. Applied Geography. 2010;30(3):303–16.

[27] Sarker BK, Rahman M, Rahman T, Hossain J, Reichenbach L, Mitra DK. Reasons for preference of home delivery with traditional birth attendants (TBAs) in rural Bangladesh: a qualitative exploration. PLoS ONE. 2016;11(1):e0146161 10.1371/journal.pone.0146161 26731276 PMC4701391

[28] Song P, Kang C, Theodoratou E, Rowa-Dewar N, Liu X, An L. Barriers to hospital deliveries among ethnic minority women with religious beliefs in China: a descriptive study using interviews and survey data. International Journal of Environmental Research and Public Health. 2016;13(8):815. 10.3390/ijerph13080815 PMC4997501 27529263

[29] Withers M, Kharazmi N, Lim E. Traditional beliefs and practices in pregnancy, childbirth and postpartum: A review of the evidence from Asian countries. Midwifery. 2018;56:158–70. 10.1016/j.midw.2017.10.019 29132060

[30] Sychareun V, Hansana V, Somphet V, Xayavong S, Phengsavanh A, Popenoe R. Reasons rural Laotians choose home deliveries over delivery at health facilities: a qualitative study. BMC Regnancy and Childbirth. 2012;12(1):86. 10.1186/1471-2393-12-86 PMC3449206 22925107

[31] Mirowsky J, Ross CE. Education, social status, and health. Newark, New Jersey: Transaction Publishers; 2003.

