# Supplementary material for: Spatiotemporal trends and ecological determinants in maternal mortality ratios in 2,205 Chinese counties, 2010–2013: A Bayesian modelling analysis
Source: PLoS Med. 2020 May 15;17(5):e1003114. doi: 10.1371/journal.pmed.1003114 (PMC7228041; doi:10.1371/journal.pmed.1003114)
Supplement: S1 Text — (DOCX) [file pmed.1003114.s001.docx]

**Study Protocol：**

**Title: Geographical Variation and Determinants of Maternal Mortality Ratio in Chinese Mainland**

**Research questions:**

1. Although China achieved a remarkable reduction of maternal mortality ratio (MMR) as one of Millennium Development Goals (MDG) in the past decades, do the spatial and temporal differences of the MMR risk existed? Specially China has an unbalanced economic and social development features. Are there some aeras with high MMR risk in western regions as the developing areas.

2. How to decrease the MMR in some developing areas of China if where high risks of MMR occurred? In other words, can the main determinants of MMR at national and subnational scale be quantified? Are the influence patterns of the MMR in various regions, e.g. developing areas vs. developed areas, different?

**Research objectives**

**Aim 1:** To explore or detect the spatiotemporal trends or patterns of the MMR in China based on surveillance data.

**Aim 2:** To identify the influencing magnitude of main determinants of the MMR at national and subnational level.

**Protocol:**

**Proposed Methods**

**Research design:** This study proposes a Bayesian statistical modelling study design of the surveillance MMR data at county level. Firstly, the space-time trends of the MMR at county level will be investigated by using a state-of-the-art Bayesian spatiotemporal model [[1](#_ENREF_1)] which can estimate the overall spatial trend, i.e. spatial relative risk, overall temporal trend, local trend, decomposed from the complicated spatiotemporal coupling process of the MMR. Furthermore, the main determinants of the MMR will also be examined. To identify the differences of the influence patterns of the MMR in various areas, the influence effects of the main determinants at national and subnational level will be estimated by using a Bayesian multivariable regression and GeoDetector model [[2](#_ENREF_2), [3](#_ENREF_3)]. We will absorb two aspects of factors or determinants, including socio-economy and medical intervention.

**Data source:** The data used in this research will include the medical dataset about the number of livebirths and maternal deaths, medical intervention for pregnant women, socio-economic data of pregnant women. The medical dataset at county-level will be collected from the National Maternal and Child Health Surveillance System (NMCHSS) over the study period. And the socio-economic data will be obtained from the China County Statistical Yearbook of the corresponding year.

**Ecological determinants:** The determinants of the MMR mainly include direct obstetric causes and indirect causes [[4-6](#_ENREF_4)]. This research mainly focuses on the non-medical causes of the MMR. One of the most important indirect determinants is the mother's income, which is a proxy of maternal health, education, malnutrition, and place of residence (urban or rural) [[7](#_ENREF_7)]. The factor of the average mother’s income in a county can be represented by the per capita income in the corresponding county. Another important indirect factor is the proportion of hospital births, which reflects the accessibility of hospitals and skilled midwives [[4](#_ENREF_4)]. The third factor is antenatal care, which helps detect obstetric diseases so that pregnant women can receive timely treatment [[7](#_ENREF_7), [8](#_ENREF_8)]. The determinant of the proportion of hospital births was quantized by the proportion of pregnant women who delivered in hospitals. The determinant of the antenatal care was quantified by the proportion of pregnant women who had five or more maternal checkups.

**Data analyses:** Our overall approach to the analysis will focus on, Bayesian spatiotemporal modelling, Bayesian multivariable model, and GeoDetector modelling. The significance of the Bayesian statistics will be directly quantified with the posterior probability of each parameter. All tests of classical statistic are at two-sided with statistical significance at P < .05.

**References:**

1. Li G, Haining R, Richardson S, Best N. Space–time variability in burglary risk: A Bayesian spatio-temporal modelling approach. Spatial Statistics. 2014;9:180-91.

2. Wang JF, Li XH, Christakos G, Liao YL, Zhang T, Gu X, et al. Geographical detectors‐based health risk assessment and its application in the neural tube defects study of the Heshun Region, China. International Journal of Geographical Information Science. 2010;24(1):107-27.

3. Wang J-F, Zhang T-L, Fu B-J. A measure of spatial stratified heterogeneity. Ecological Indicators. 2016;67:250-6.

4. Graham WJ, Witter S. Counting what counts for maternal mortality. The Lancet. 2014;384(9947):933-5.

5. Khan KS, Wojdyla D, Say L, Gülmezoglu AM, Van Look PF. WHO analysis of causes of maternal death: a systematic review. The Lancet. 2006;367(9516):1066-74.

6. El Arifeen S, Hill K, Ahsan KZ, Jamil K, Nahar Q, Streatfield PK. Maternal mortality in Bangladesh: a Countdown to 2015 country case study. The Lancet. 2014;384(9951):1366-74.

7. Chowdhury ME, Botlero R, Koblinsky M, Saha SK, Dieltiens G, Ronsmans C. Determinants of reduction in maternal mortality in Matlab, Bangladesh: a 30-year cohort study. The Lancet. 2007;370(9595):1320-8.

8. Richardson S, Thomson A, Best N, Elliott P. Interpreting posterior relative risk estimates in disease-mapping studies. Environmental Health Perspectives. 2004;112(9):1016-25.
