# Supplementary figures and images for: Spatiotemporal trends and ecological determinants in maternal mortality ratios in 2,205 Chinese counties, 2010–2013: A Bayesian modelling analysis
Source: PLoS Med. 2020 May 15;17(5):e1003114. doi: 10.1371/journal.pmed.1003114 (PMC7228041; doi:10.1371/journal.pmed.1003114)

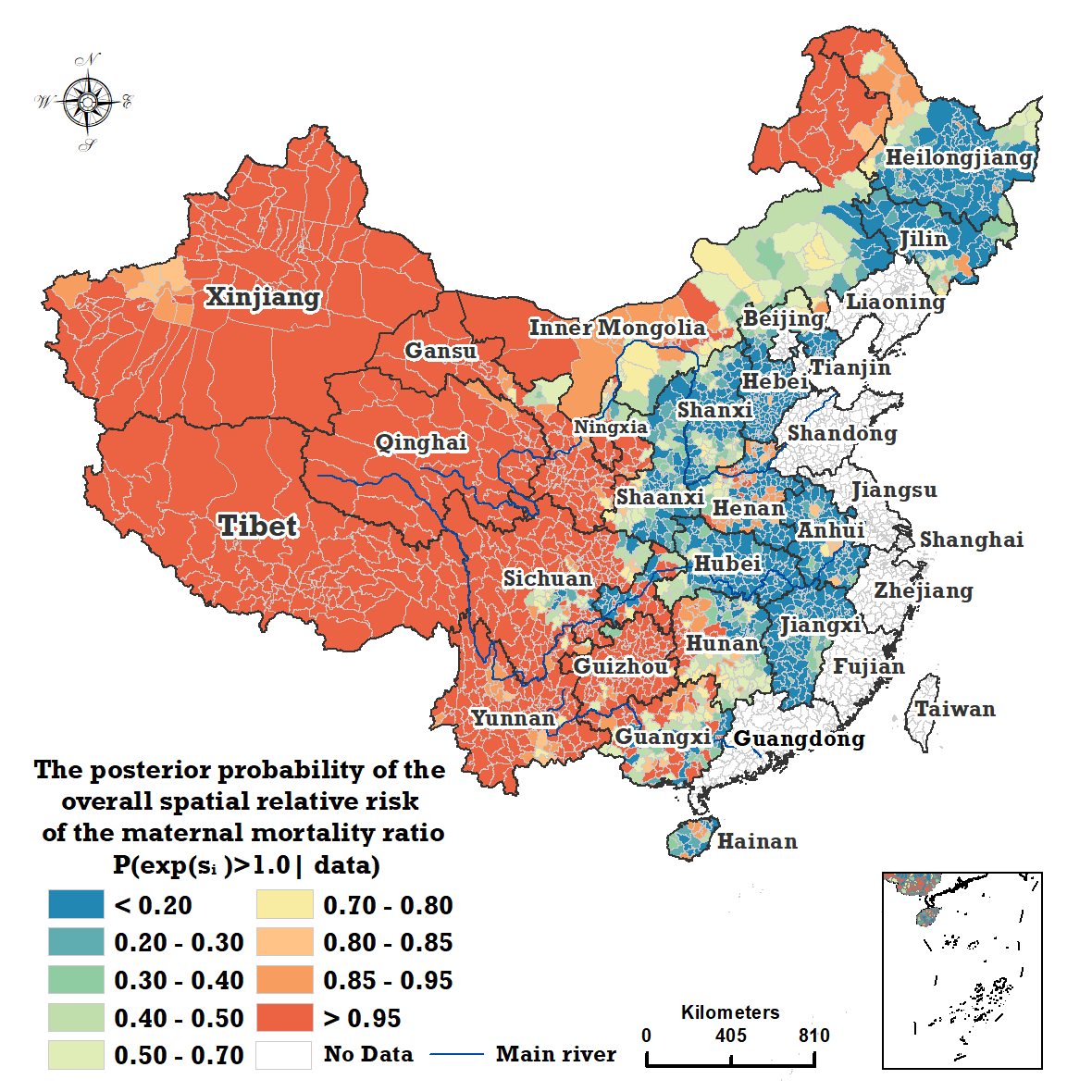

Supplement: S1 Fig — MMR, maternal mortality ratio. (TIF) [file pmed.1003114.s003.tif]

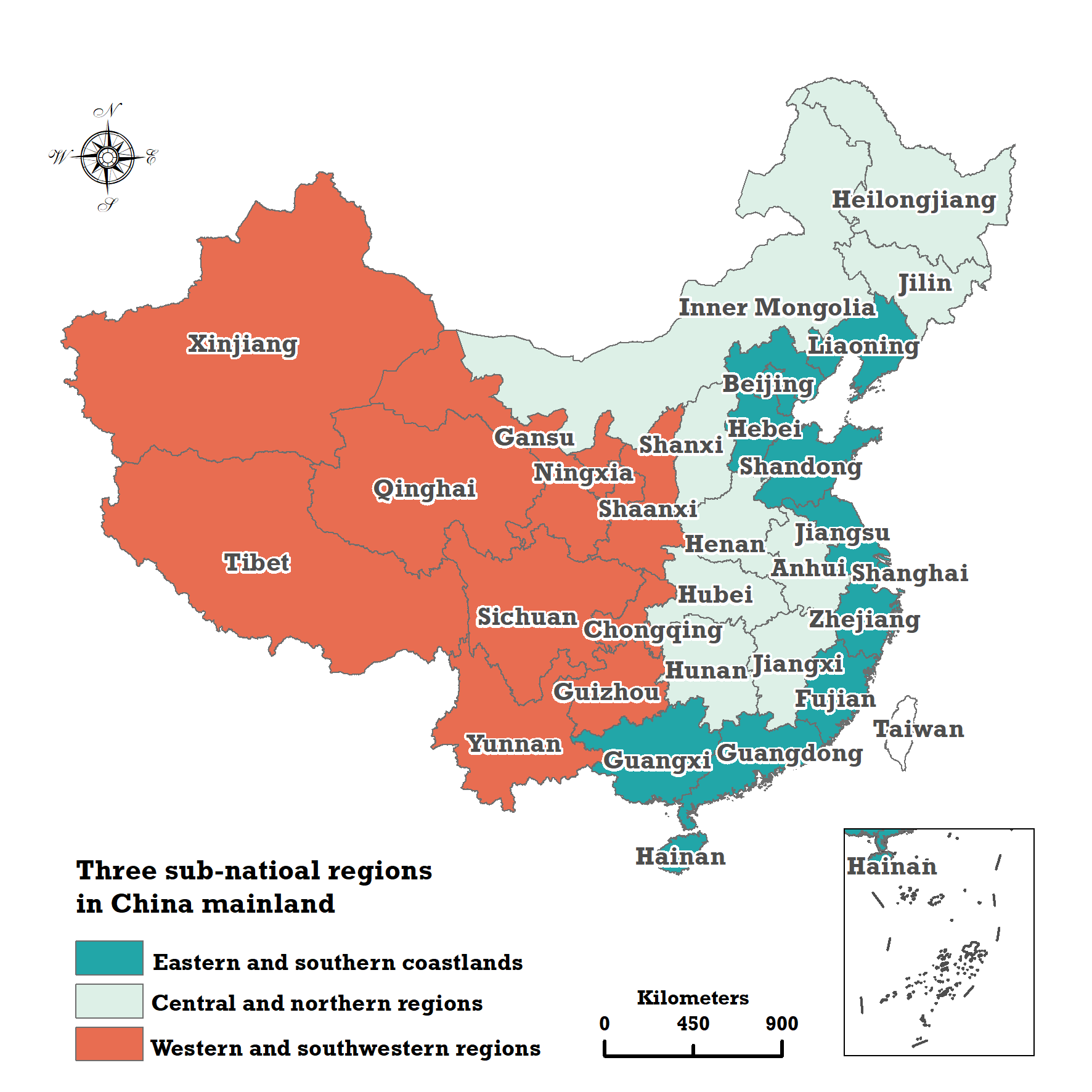

Supplement: S2 Fig — (TIF) [file pmed.1003114.s004.tif]
